# A renewed call for transdisciplinary action on NCDs

**DOI:** 10.1186/s12914-020-00241-z

**Published:** 2020-08-28

**Authors:** Brigit Toebes, Marlies Hesselman, Jochen O. Mierau, Jitse P. van Dijk

**Affiliations:** 1 Global Health Law Groningen Research Centre, Department of Transboundary Legal Studies, Department of International Law, Faculty of Law, Aletta Jacobs School of Public Health, University of Groningen, PO Box 716, 9712 EK Groningen, the Netherlands; 2 Department of Transboundary Legal Studies, Faculty of Law, University of Groningen, Oude Kijk in‘t Jatstraat 26, 9712 EK Groningen, the Netherlands; 3 Aletta Jacobs School of Public Health & Faculty of Economics and Business, University of Groningen, Nettelbosje 2, 9747 AE Groningen, The Netherlands; 4 Department of Community and Occupational Medicine, University Medical Center Groningen, University of Groningen, Ant. Deusinglaan 1, 9713 AV Groningen, the Netherlands

## Abstract

Notwithstanding COVID-19, non-communicable diseases (NCDs) will be the leading cause of death in every region in the world by 2030. This contribution, which forms an introduction to our collection of articles in this journal, identifies elements for a transdisciplinary research agenda between law, public health, health economics and international relations aimed at designing concrete interventions to curb the NCD pandemic, both globally and domestically.

## Introduction

Three years ago we called in this journal for progress on an interdisciplinary research agenda for law and policy interventions to cope with curbing the non-communicable disease (NCD) pandemic [1]. Specifically, we argued in favour of an interaction between law, public health, health economics and international relations aimed at designing concrete interventions to curb the NCD pandemic, at national and international level.

By 2030, NCDs will be the leading cause of death in every region in the world [2]. Much of the global NCD burden (40%) is linked to four “modifiable behavioral risk factors” that affect many countries: tobacco use, unhealthy diets, physical inactivity and harmful use of alcohol [2]. In this context, there is an association between socio-economic inequalities, NCDs and these risk factors for NCDs [3]. The scale of the problem makes NCDs a pandemic phenomenon that requires a powerful international and domestic response.

Researchers from various disciplinary angles have engaged with NCDs and made a meaningful contribution to the development of effective NCD laws and policies [4–7]. However, we observe that interdisciplinary research geared at law and policy-making does not always receive the support and outreach it deserves. Specifically, many more efforts and resources should be allocated to interdisciplinary research endeavours, with implications for law and policy-making, which, given the magnitude and complexity of the problem, deserve more attention.

In our Debate piece published in 2017 in this journal we identified a number of gaps in current research and called for an interdisciplinary research agenda between law and other disciplines aimed at designing concrete proposals for laws and policies to curb the NCD pandemic, both globally and domestically [1]. In a subsequent call for papers we invited scholars from various health-related disciplines to submit a paper focusing on the identification of this research agenda with the aim of identifying concrete solutions to the NCD pandemic. The result of this call for papers is the present collection of articles in BMC International Health and Human Rights, herewith launched.

This introductory paper sets the stage for this collection of papers and identifies research approaches from four disciplinary angles: public health, health economics, law, and international relations. Through an interaction between these approaches, we aim to draw some conclusions on these disciplinary dimensions and their interaction. We recognise that this selection is by no means exhaustive of all the disciplinary dimensions to the debate. The overall goal of our proposed integrated research agenda is to fill the policy ‘toolbox’ of governments with evidence-based proposals for sound and effective law and policy interventions on curbing NCDs. This type of research needs to be developed in particular in Low and Middle Income Countries (LMICs), where little research on NCDs is carried out, in particular when it comes to evaluating good practice interventions in a local context [7].

## Public health: quantifying the burden and modifiable risk factors

The first thing that should happen from a public health perspective, to prevent the creation of ‘solutions without problems’, is to quantify the disease burden and to understand the key drivers of NCDs. The Global Burden of Disease-group (GBD-group) has recently published its findings on disability-adjusted life-years (DALY) and healthy life expectancy on a global, regional and national scale, for a very large number of diseases, including specifically NCDs [8]. The same GBD-group carried out a global, regional, and national comparative risk assessment of a large number of risk factors in all countries of the world [9]. Their study found that out of 34.1 million deaths, 61% could be attributed to risk factors covered by the GBD study, and 48.3% of 1.21 billion DALYs. In terms of leading risk factors, high systolic blood pressure (SBP) ranked highest (accounting for 10.4 million deaths and 218 million DALYs), followed by smoking (7.10 million deaths and 182 million DALYs), high fasting plasma glucose (6.53 million deaths and 171 million DALYs) and high body-mass index (4.72 million deaths and 148 million DALYs). In 2017, this high SBP was the leading Level 4 risk factor for age-standardised DALY rates in four super-regions: Central Europe, Eastern Europe, and Central Asia; North Africa and Middle East; South Asia; and Southeast Asia, East Asia, and Oceania. Instead, smoking was the leading risk factor in the high-income super-regions (Europe and North America), and high BMI in the Latin America and Caribbean region. Understanding key drivers of NCD risk is essential for successfully curbing their incidence, and related health risks.

A next step would be forecasting life expectancy, years of life lost, and all-cause and cause-specific mortality for a large number of diseases in a large number of countries and territories with reference and alternative scenario’s [10]. A further related step would be the question where we are now in curbing NCDs and what should be done [11].

The WHO currently identifies four NCDs (cardiovascular diseases, cancer, diabetes and chronic respiratory diseases) as the leading cause of death, with NCDs generally being responsible for 70% of deaths world-wide. It also identifies four key modifiable risk factors that contribute to these four diseases (tobacco use, unhealthy diet, lack of physical activity, and the harmful use of alcohol). According to the WHO, these factors inter alia “lead to overweight and obesity, raised blood pressure, and raised cholesterol, and ultimately disease” [12].

The WHO and the international community, including through UN Sustainable Development Goal 3.4, have committed to curb the global NCDs epidemic and especially to reduce premature mortality from NCDs through prevention and treatment [13]. As it is difficult to decrease mortality due to NCDs *directly*, it seems easier to *indirectly* decrease mortality by influencing the modifiable behavioural risk factors, like tobacco use, unhealthy diet, lack of physical activity, and the harmful use of alcohol. These behaviours in turn lead to pathological conditions contributing to NCDs.

WHO has called these modifiable behavioural risk factors a ‘best buy’. In 2017 and 2020, the WHO has monitored per country the revised set of WHO ‘best-buys’ and other recommended interventions for the prevention and control of NCDs, also endorsed by the World Health Assembly in May 2017 [12]. The ‘best buys’ currently consist of 16 practical and cost-effective interventions that can be delivered at the primary level and include measures like increasing tobacco taxes; restricting alcohol advertising; reformulating food products with less salt, sugar and fat; vaccinating girls against cervical cancer; treating hypertension and diabetes; and more. They are presented as ‘a powerful economic tool’ in that the WHO estimates that every dollar invested in them ‘will yield a return of at least seven dollars’, as well as save 10 million lives by 2025, and prevent 17 million strokes and heart attacks by 2030 [14]. More expertise needs to be generated as to how these best buys can best be transformed into domestic law and policies, in light of regional and domestic circumstances and demands.

## Health economics

The field of economics has several novel contributions to make to the toolbox for policy interventions, especially in terms of novel ways with which, in the absence of randomized controlled trials, something can be said about the causal relationship between a set of measurable quantities, like having suffered from hunger and health outcomes later on in life [15]. From a policy perspective, knowledge of such empirical relationships is crucial as it assists in developing policies that target the mechanisms by which (adverse) health outcomes come about and can contribute to the reduction of the NCD burden [16].

A good illustration is offered by the early-life determinants of chronic disease burden later in life [17]. Many studies have, and are still, associating adverse early life conditions with (chronic) health outcomes in later years [18]. Generally, such studies highlight that children who, for instance, grew up in low-income households go on to develop a host of health conditions. It is tempting to design policies aimed at raising income standards, yet the association only tells half the story. After all, the question remains whether parents who “generate” a low-income household simply also “generate” children who go on to develop all kinds of health problems, regardless of the low-income household in which they grew up. In that case boosting incomes - through transfers or subsidies - would not achieve the desired health effect.

Thus, what can, and has been, done? Economists like to seek out natural experiments - situations in which the environment around us provides us with a near experimental setting. The fluctuations of the economy provide such natural experiments [19]. Indeed, comparing two cohorts, one born during a recession and one just before or after, provides us with a treatment group (recession babies) and a control group (babies born in more tranquil times). The information load is quite low, a date and place of birth suffice, the costs are marginal and there is no ethical component as the researcher himself or herself did not cause the recession. These cohorts would then be checked against prevalent health outcomes and the analysis can be performed. Working along these lines, a host of health economic studies have highlighted that adverse economic conditions early in life causally affect health outcomes during a person’s life course. For instance, on birth weight [20], on cardiovascular disease risk [21], on all-cause mortality [22], on dementia [23] and on many others. These results call for a strong focus on conditions early in life for the prevention of disease late(r) in life.

The above approach focusing on incidents earlier in life and on health outcomes later in life, illustrates how health economics can assist in the quest to reduce the burden of NCDs by unraveling the causal mechanism that lie underneath the rising NCD epidemic. Most of the time, the required data already exist.

## International relations

As evidence on the NCD disease burden and risk factors are built, relevant national and international laws and policies will be necessary to curb, steer, forbid or stimulate any (un)desirable behaviour.

Especially in the sphere of NCDs, risk factors are not affected only by individual behaviour, but also heavily impacted by the (contradictory) interests and agendas of major powerful actors such as tobacco, food and beverages, or pharmaceutical industries [24]. For such reason, sufficiently powerful responses, including in the form of binding regulations, are necessary. Concerns have been voiced in particular about the spread of NCDs to and in developing countries, and the truly global reach of some multi-national companies marketing harmful products to (vulnerable) customers. Tackling NCDs globally, therefore, does not just require action by individual countries, but international cooperation and sharing of (best) practices, including for example, on the most effective implementation of the WHO’s ‘best buys’.

There is already a strong international cooperative practice available through the first binding treaty on NCDs: the Framework Convention on Tobacco Control under auspices of the WHO in 2005. Important lessons can be drawn from this effort (see further below) [25]. In fact, over the years, several so far unsuccessful proposals have been made to replicate the FCTCs regulatory initiative, such as the calls for a Global Convention on Healthy Diets from 2014 onwards, in response to the perceived failure of the WHO’s Healthy Diets Strategy adopted 10 years prior [26], or the failed initiative for a Global Alcohol Convention in 2006.

Our article of 2017 suggested that successful law and policy-making in the sphere of NCDs will benefit from further understanding and insights into how international (legal) norms may emerge over time, or be met with resistance [1, 25]. In particular, research at the intersections of international law and international relations will improve our understanding of how formulation and adoption of new legal norms may be shaped through appropriate framing of problems, solutions and the consistent spread of public messages on NCDs. Or similarly, may require suitable, powerful ‘norm entrepreneurs’ with relevant organizational platforms in early phases of norm development [27, 28]. While the success of any normative campaign will depend on many different factors, it is argued that further research into the (successful) framing and lobbying by anti-tobacco interests groups, as well as key figures and key countries, may yield valuable insights into how relevant actors in this sphere worked together successfully, or were supported or thwarted in their efforts to achieve (strong) evidence-based law and policy-intervention.

In recent years, several (comparative) works on global network-formation around tobacco, alcohol and other risk factors were published to shed light on such questions [29, 30]. Such works have *inter alia* suggested that the anti-tobacco movement was exceptionally effective because they developed effectively ‘from a group of dedicated individuals into a strong international coalition’ backed by membership-based organizations (achieved ‘network-formation’), and also succeeded in formulating and maintaining a widespread consensus about both the dangers of tobacco, along with the requisite policy measures to control its use (achieved ‘issue-formulation’ and fostered ‘scientific closure’ on tobacco harms) [29, 30]. Instead, alcohol control organizations are much more diverse and loosely organized group(s) of organisations, advancing different problems and solutions around alcohol as key concerns, e.g. in the sphere of treating addition, NCD incidence, alcohol-related violence, or public health issues, including road-safety [29, 31]. In short, the alcohol movement may so far lack the same dedicated ‘coalition-forming’ or ‘network formation’, enabling them to formulate a sufficiently effective and compelling global message on how and why it is important to more strictly regulate alcohol consumption, in specific manners.

Overall, such research on the emergence, adoption and implementation of new global (legal) norms can be found at the intersections of the disciplines of law, international relations, and political science, and includes useful analytical models such as ‘norm-life cycle models’, ‘norm spiral models’, or theories about ‘transnational legal processes’ or the role of ‘transnational advocacy networks’, pushing for global norms, but also bringing them home and translating them into domestic systems [32, 33].

## Law as a tool to reduce risk factors

Research supports that law is an important tool to reduce modifiable behavioural risk factors. A range of proven effective legal tools are at the disposal of governments to reduce smoking, excessive use of alcohol, and the consumption of unhealthy diets. The above-mentioned ‘best buys’ as developed by WHO offer guidance, as well as a vast amount of scientific research investigation on the effectiveness of these legal tools. Such evidence is most prevalent and convincing in relation to tobacco use and exposure to second-hand smoke (SHS). For example, there is ample research underlining that tobacco taxes reduce tobacco consumption [34], as well as research proving the beneficial health effects of smoke-free zones [35]. There are, furthermore, some positive experiences with taxes on sugar-sweetened beverages [36] and marketing of unhealthy food products [37].

While legal action on NCDs ultimately has to be implemented by domestic legal systems, adapted to local circumstances, there is international and regional legislation that informs domestic states how to regulate the modifiable risk factors. Again, this legislation is most developed when it comes to tobacco, through the FCTC. This treaty, that has been ratified by 181 States, sets standards in relation to, *inter alia*, the interaction between government and the tobacco industry, price and tax measures, and protection from exposure to tobacco smoke [38]. It has had considerable impact on domestic tobacco legislation, in the sense many States Parties have amended their domestic tobacco legislation in order to comply with the treaty [39]. Many countries, however, are lagging behind in their implementation of international commitments, [40, 41], while other States go beyond the FCTC by adopting further reaching measures, for example by prohibiting smoking in cars when a child is on board [42]. Legal researchers have increasingly begun to analyse what inspires legal compliance or ambitions, and in any case, the reasons or mechanisms for (non) implementation or (lack of) ambition deserves further understanding. Legal research will benefit here from collaborative research with other disciplines, such as international relations, political science, or sociology.

Regulating risk factors touches on a range of values that are inherent in individual dignity. Such values are also protected by law in the form of international and regional human rights standards that provide a legally binding framework for protecting them, as well as for balancing the various interests involved [43]. On the one hand, rights to life, health and information for example reflect governmental duties to take measures to protect individuals against the harms caused by risk factors. On the other hand, measures aimed at modifying behaviours may imply restrictions of the full freedom someone has – or at least people perceive to have – including interventions ‘behind the front door’. Here human rights to physical integrity, privacy and freedom of movement come into play. A balancing act between the protection of health and individual freedom does not always lead to a clear outcome. Yet it is important to articulate such values, make them visible, and take them into account when taking NCD control measures. More efforts can be made to identify so-called ‘human rights based approaches’ toward reducing risk factors [33]. Specifically, a ‘child rights’ approach gives guidance into how the best interests of children can be protected in the context of the mentioned risk factors [44].

Finally, human rights law is also a useful framework for addressing socio-economic conditions that go beyond behavioural risk factors. The human rights framework reflects the notion that a broad range of social determinants are crucial for the achievement of good health [45, 46]. Importantly, human rights law does not only inform sound law and policy making, but is an important vehicle for accountability of governments and powerful actors, and can inform agenda-setting. In what contexts human rights should and can be effectively harnessed is an important aspect of the NCD research agenda.

## Concluding observations

We argue that solutions to the NCD pandemic can be found, or must necessarily be found, through an interaction between various disciplines, including the ones we identify in this piece. While public health and health economics identify the data on which new laws and policies should rest, international relations theory may help understand or identify the political processes through which new laws and policies can be promoted and adopted, or the types of obstacles or resistance that could be expected. Through this interaction, sound NCD laws, policies and other interventions can be identified that benefit individuals, paying due respect to their human rights.

Our overview has also revealed some gaps in existing research. From a public health perspective, more data can be generated on the disease burden and on modifiable risk factors. Economics can enhance our understanding of the causal impact of risk factors on health outcomes as well the impact of various laws, policies and interventions aimed at curbing the NCD epidemic. From an international relations and legal perspective, the feasibility and nature of international and domestic laws and policies regulating alcohol and diets could be further explored, especially in light of what is perceived as (relative) success of the anti-tobacco movement, as well as the role and potential of human rights in regulating risk factors. Adequate implementation of laws and policies will raise a further range of questions, not addressed so much in this Issue, including questions of tailoring interventions to evidence and concerns in specific domestic contexts, as well as ensuring adequate implementation, compliance and accountability. In this sense, we highlight especially the importance of fostering the right culture, values and mechanisms for accountability for violations of NCD laws and policies, and of human rights, including by industry [47].

## Supplementary information

**Additional file 1.**
